# Diffuse large B-cell lymphoma in the uterus with unexpected manifestations: a case report

**DOI:** 10.1186/s13256-024-04657-2

**Published:** 2024-07-16

**Authors:** Kenana Tawashi, Tuka Hamasho, Mustafa Al-Hussien, Ameen Ammar

**Affiliations:** 1 Hama University, Faculty of Medicine, Hama, Syria; 2 Al Asad Hospital, Hama, Syria

**Keywords:** Non-Hodgkin lymphoma, Uterus, Diffuse large B-cell lymphoma

## Abstract

**Background:**

Lymphoid neoplasm is a common disease, arising from lymphoid cells. It is divided into Hodgkin lymphoma and non-Hodgkin lymphoma. Non-Hodgkin lymphoma can be intranodular or extranodular, which happens in 25% of primary cases. The most common locations of extranodular non-Hodgkin lymphoma are the skin and gastrointestinal tract. The genital tract is a rare location; most lymphomas arise from the cervix and vagina, while the uterine corpus is an extremely rare location. In our case, the patient was diagnosed with primary extranodular non-Hodgkin lymphoma in different locations of her genital tract.

**Case presentation:**

A 48-year-old nonparous Syrian woman complained of diffuse abdominal pain, fatigue, debility, high fever, vomiting, and urinary retention for a week. The last menstrual period of the patient was 5 years previously. The physical examination showed periodic abdominal pain with severe fatigue and increased abdominal size. The laboratory investigations were within normal limits except for a low level of hemoglobin and a high level of cancer antigen 125. The radiological investigations showed a uterine sizable lobulated mass with irregular borders and high and heterogeneous density, extending to the right and left ovaries, enlargement lymph nodes around the abdominal aortic and right iliac vessels, and severe right pleural effusion with right inferior lobe atelectasis. A total hysterectomy and oophorectomy were done. The histopathological examination showed that the patient had non-Hodgkin lymphoma (primary tumor).

**Conclusion:**

Primary non-Hodgkin lymphoma in the female genital tract is an extremely rare disease. Fast diagnosis and treatment can improve the outcomes, so this differential diagnosis should be in our minds even in the absence of systematic manifestations of lymphoma. More studies are needed to explain the pathology of this disease and to put guidelines that determine the perfect methods for diagnosis and treatment.

**Supplementary Information:**

The online version contains supplementary material available at 10.1186/s13256-024-04657-2.

## Introduction

Lymphoid neoplasm is a common disease, arising from lymphoid cells, which are an important component of the immune system and play an essential role in the body’s defenses [1]. Lymphomas are divided into Hodgkin lymphoma (HL) and Non-Hodgkin lymphoma (NHL). HL, which forms 10% of all lymphomas, is divided into classical and nonclassical types, while NHL, which forms 90%, is divided into B-cell, T-cell, and natural killer (NK) cell types [2]. For most categories, lymphomas are more common in men [1]. Many risk factors are identified such as (1) immune disorders, which may be genetic or acquired (severe combined immunodeficiency); (2) medicines that affect the immune system such as tumor necrosis factor-alpha inhibitors; (3) infections such as *Helicobacter pylori*, *Campylobacter jejuni*, human T-cell lymphotropic virus, hepatitis C, human herpes virus-8, and human immunodeficiency virus; and (4) occupational factors such as herbicides and pesticides [2, 3]. NHL can be intranodular or extranodular, which happens in 25% of primary cases [4]. The most common locations of extranodular NHL are the skin and gastrointestinal tract [5]. The genital tract is a rare location (only about 2% of cases); most lymphomas arise from the cervix and vagina, while the uterine corpus is an extremely rare location [6]. In our case, the patient came with unspecific manifestations and she was diagnosed with primary extranodular NHL in different locations of her genital tract.

## Case presentation

**Fig. 1:**
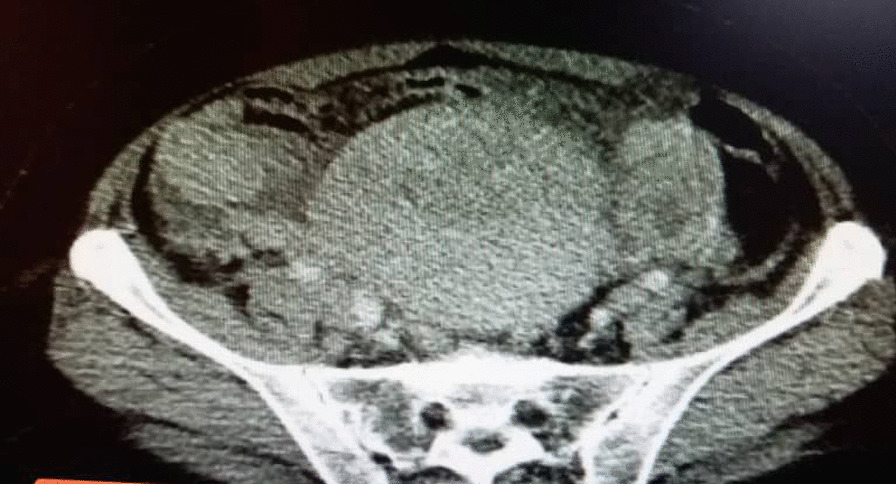
The computed tomography of the chest, abdomen, and pelvis after administering intravenous contrast agents showed a uterine sizable lobulated mass with irregular borders and high heterogeneous density, measuring about 112 mm × 191 mm × 160 mm, extending to the right and left ovaries

**Fig. 2:**
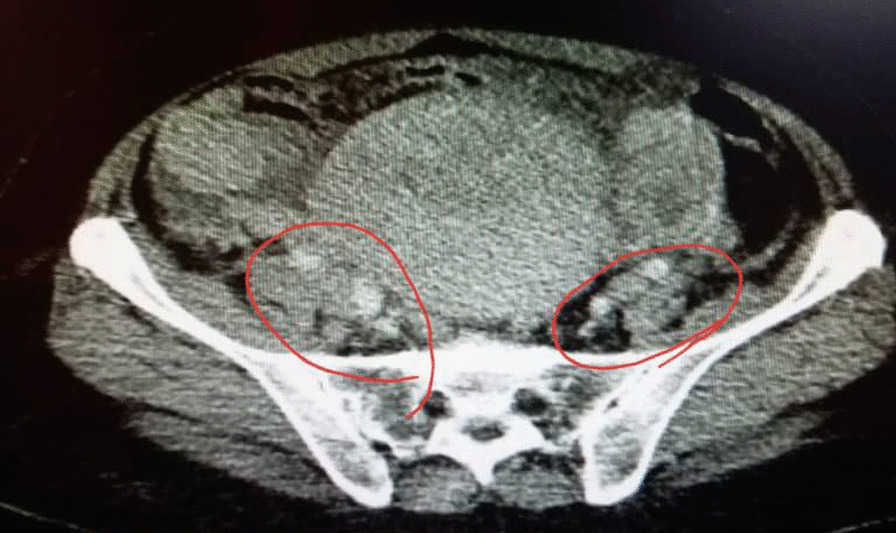
The computed tomography showed also enlargement lymph nodes around the abdominal aortic and right iliac vessels with signs of mesenteric metastases and moderate ascites. The red circles reveal the lymphadenopathy

**Fig. 3:**
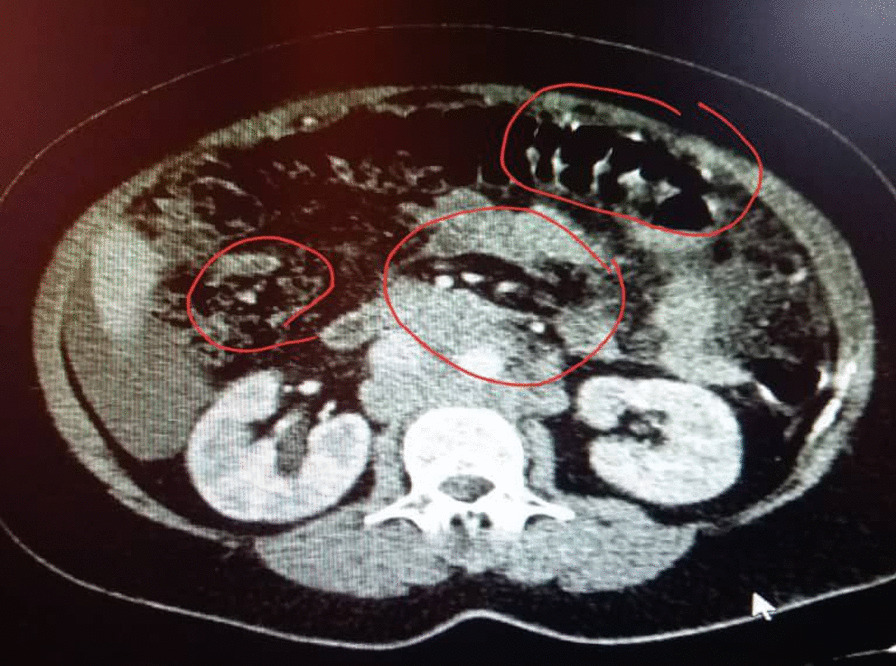
The computed tomography showed also enlargement lymph nodes around the abdominal aortic and right iliac vessels with signs of mesenteric metastases and moderate ascites. The red circles reveal the lymphadenopathy

**Fig. 4:**
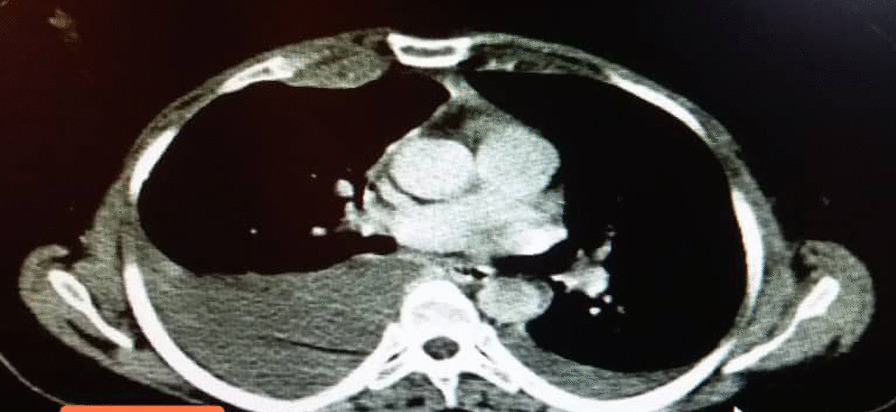
The computed tomography showed a severe right pleural effusion with right inferior lobe atelectasis

**Fig. 5:**
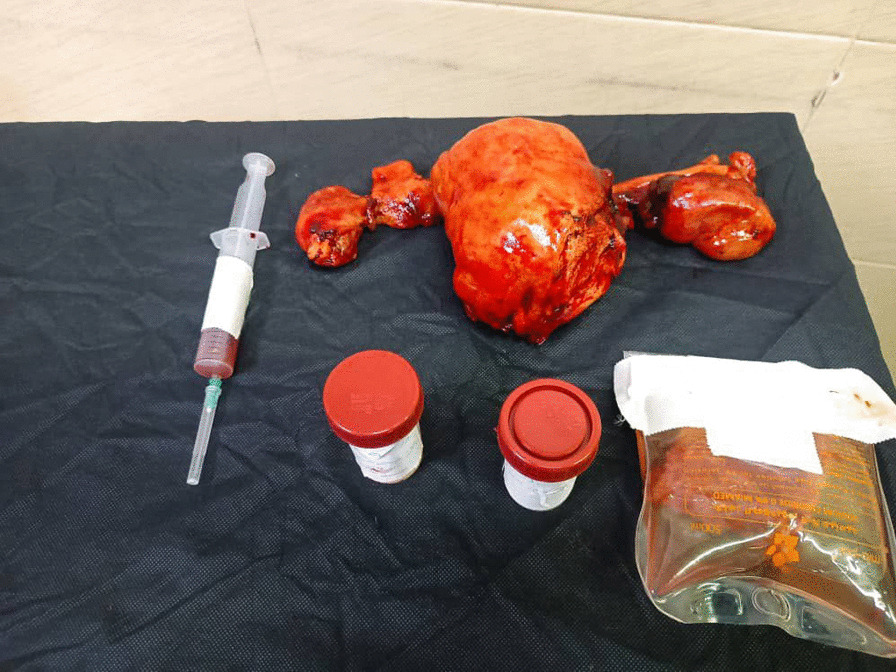
Photos from the surgery

A 48-year-old nonparous Syrian woman came to the emergency department complaining of diffuse abdominal pain, fatigue, debility, high fever, vomiting, and urinary retention for a week. The patient’s medical history revealed that she was diagnosed with cystitis and treated by the area’s doctor with no response after 3 days of treatment. The surgical history revealed that she underwent hemorrhoid excising. The drug, family, and psychosocial histories were unremarkable. The last menstrual period of the patient was 5 years earlier. The physical examination showed periodic abdominal pain that differed in severity (from mild to severe) for a week with severe fatigue and increased abdominal size. There were no night sweats or weight loss. The examination of the other systems was normal. The laboratory investigations were within normal limits, except a low level of hemoglobin (11 mg/dl) (reference values 12–14 mg/dl) and a high level of cancer antigen (CA)-125 (333.75 U/ml) (reference values 1.7–32 U/ml). The ultrasound imaging showed a heterogeneous lobulated mass on the anterior and superior walls of the uterus, extending to the right and left ovaries; with high and low echoes inside it. The echo Doppler of the mass showed high perfusion. In addition, there was moderate free liquid in the abdomen. The computed tomography of the chest, abdomen, and pelvis after administering an intravenous contrast agents showed a uterine sizable lobulated mass with irregular borders and high and heterogeneous density, measuring about 112 mm × 191 mm × 160 mm, extending to the right and left ovaries (Fig. 1). It also showed enlargement of lymph nodes around the abdominal aortic and right iliac vessels with signs of mesenteric metastases and moderate ascites (Figs. 2, 3); in addition, there was a severe right pleural effusion with right inferior lobe atelectasis (Fig. 4). After explaining the situation to the patient and obtaining her written consent, we decided to do a hysterectomy. A cardiac consultation was taken before the surgery and was within normal. A total hysterectomy and oophorectomy were done by a specialist in obstetrics and gynecology (Fig. 5). We took biopsies from the uterus, ovaries, peritoneum, and free liquid in the abdomen. The patient's situation was good after the surgery. The histopathological examination of the excised biopsies showed that the patient had NHL (primary tumor); a diffuse large B-cell lymphoma with high-grade Burkitt-like features was observed. The immunohistochemistry examination showed that the tumor was CD20^+^, CD3^−^, and Ki-67 positive in 80% of the cells (supplement 1). An oncology consultation was taken but the patient did not receive any treatment because she died suddenly after 20 days of surgery by pulmonary embolism.

## Discussion

Lymphoma is one of the top ten most common cancers in the world; it is a well-known neoplasm that arises from immune cells, which are an important component of the body’s defenses [1]. Lymphomas are divided into HL and NHL. NHL, which forms 90% of all lymphomas, is also divided into B-cell, T-cell, and natural killer cell lymphomas [2]. The most common type is diffuse large B-cell lymphoma (DLBCL), which is a subtype of B-cell lymphoma. It can be intranodular (60%) or extranodular (40%) and is considered the most common lymphoma that arises in extralymphatic locations (the gastrointestinal tract is the most common location followed by bone and central nervous system) [2, 7]. Another subtype of B-cell lymphoma is Burkitt lymphoma, which is divided into endemic, spontaneous, and immunodeficiency-associated [2]. A subtype of lymphomas is called a double-hit lymphoma, which forms when it contains MYC and either BCL2 or BCL6 mutations; this subtype is often seen in patients who have disease at the interference between Burkitt lymphoma and DLBCL [3]. The histological examination of the biopsies in our case revealed DLBCL with high-grade Burkitt-like features, which may be a double-hit lymphoma, but because of the lack of resources in our country, we could not do a karyotype. Many factors encouraged us to think about this unique type of lymphoma (the unexpected location and unspecific manifestations). Even NHL can form in any part of the body; the genital tract is an extremely rare location (about 2%) [6]. Some studies link lymphomagenesis and high exposure to female sex hormones for a long time, and they also considered pregnancy a protective factor because it changes the concentration of the female sex hormones and the efficacy of the immune system, while other studies showed that there is not any relationship; the patient in our case did not experience pregnancy, which may play a role in the lymphomagenesis [1]. Patients with NHL can present with systematic symptoms and signs such as fevers, severe night sweats, pruritus, weight loss, fatigue, and painless lymphadenopathy. As NHL can form in any part of the body, the clinical manifestations vary a lot depending on the location [3]. When NHL forms in the genital tract, the symptoms include bleeding, abdominal or pelvic pain or discomfort, back pain, and abdominal or pelvic masses [6, 8]. The patient in our case came with fatigue, abdominal pain, vomiting, and urinary retention, which are unspecific symptoms (systematic and volumetric). The differential diagnosis of NHL in the genital tract includes chronic cervicitis, small-cell carcinomas, adenocarcinomas, and cervix sarcomas when the lesion forms in the cervix, but when it forms in the uterus the differential diagnosis includes sarcomas, reactive inflammatory lesions, typical uterine leiomyomas with massive lymphoid infiltration, and inflammatory pseudotumors of the uterus [6, 7]. Many factors make the diagnosis of NHL in the female genital tract very difficult such as unspecified manifestations, vague findings in physical examination, and its rarity in this location [6]. Many laboratory and radiological methods help us in the diagnosis of lymphoma but tissue biopsy is still the cornerstone [2]. Every diagnosed lymphoma should be staged before initiation of the treatment; the staging is done depending on (1) the patient's history and examination, (2) laboratory investigations such as basic blood work (including lactate dehydrogenase) and bone marrow biopsy, (3) radiological investigations such as whole body positron emission tomography or computed tomography, and (4) cerebrospinal fluid testing in some special cases [2, 3]. Some studies emphasized that magnetic resonance imaging is better than computed tomography in determining the disease’s nature and the extent of the tumor [6]. The Ann Arbor Staging System is the most used one (Table 1) [2]. Other studies described criteria to help physicians in the diagnosis of primary uterus lymphoma, including (1) no evidence of leukemia depending on the peripheral blood cells, (2) the investigations emphasized that there is not any other affected location in any part of the body, (3) the disease is limited to the uterus at the time of diagnosis, and (4) if any other tumor is revealed and removed from the genital tract; an interval of several months must have elapsed between the diagnosis of the primary and secondary tumors [8]. The treatment choices include surgery, radiotherapy, and chemotherapy. Surgery is used in localized conditions (hysterectomy in cases such as ours) [6]. Radiotherapy also is considered an adjuvant treatment with chemotherapy to achieving long-term remission; it works by damaging the affected organs and it is considered essential in many cases such as when tumors do not respond to chemotherapy or respond partially and patients who cannot take chemotherapy because of its complications [7]. Chemotherapy is the golden standard in treating uterus NHL; six cycles of cyclophosphamide, vincristine, doxorubicin, rituximab, and prednisone (R-CHOP) are given, and the interval period between each one is about 3 weeks [2, 3]. The patient in our case underwent a hysterectomy, which was done to help in making the diagnosis, but she did not receive any special treatment for the NHL. The follow-up is essential in such patients; it contains history, physical examination, and laboratory investigations, while radiological investigations are needed in specific situations. The interval period between each visit increases after 5 years of remission because most relapsed cases happen in the first 2 years [2].

**Table 1:** The Ann Arbor staging system

Stage	Definition
I	The lesion includes one lymph node region or lymphoid structure such as the thymus, Waldeyer’s ring, and spleen
II	The lesions include two or more lymph node regions or lymphoid structures on the same side of the diaphragm
III	The lesions include two or more lymph node regions or lymphoid structures on the two sides of the diaphragm
IV	Extralymphatic location involvement such as bone marrow or liver
A	There are none of any one of these manifestations: (1) fever more than 38 °C (which happened two times or more in 1 month), (2) severe night sweats (in the last month), (3) weight loss for unknown reasons including 10% or more from the body weight during the last 6 months
B	Any one of the mentioned manifestations on the A group
E	Involvement of only one extralymphatic tissue except bone marrow or liver

## Conclusion

Primary NHL in the female genital tract is an extremely rare disease. Fast diagnosis and treatment can improve the outcomes, so this differential diagnosis should be in our minds even in the absence of systematic manifestations of lymphoma. More studies are needed to explain the pathology of this disease and to put guidelines that determine the perfect methods for diagnosis and treatment.

### Supplementary Information

Supplementary Material 1. The histopathological examination of the excised biopsies.

## Data Availability

Not applicable.
