# Supplementary figures and images for: Diffuse large B-cell lymphoma in the uterus with unexpected manifestations: a case report
Source: J Med Case Rep. 2024 Jul 16;18:325. doi: 10.1186/s13256-024-04657-2 (PMC11251107; doi:10.1186/s13256-024-04657-2)

Supplement 1:


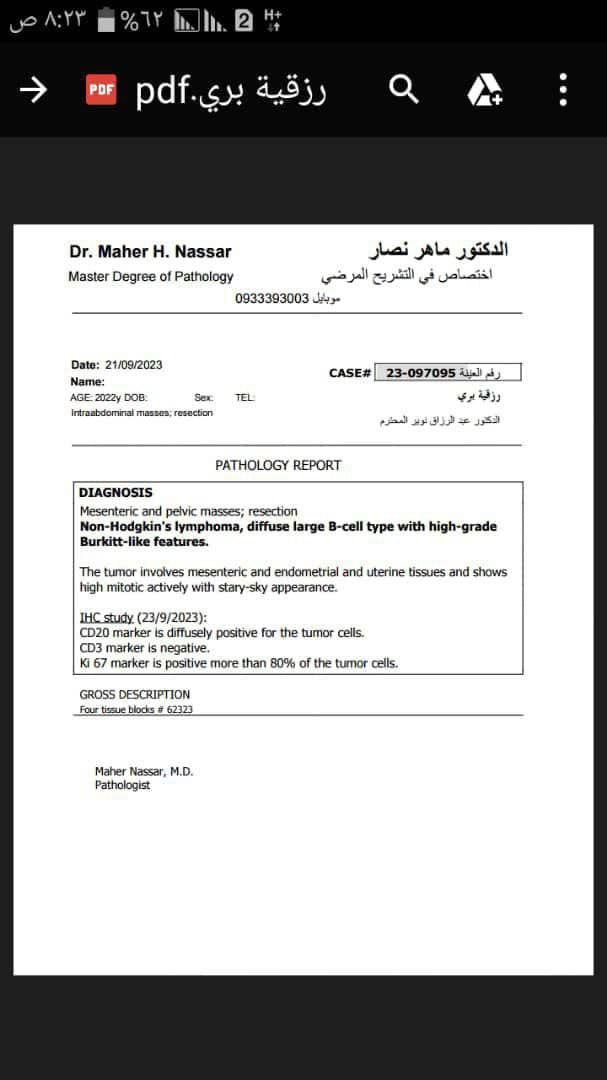
The histopathological examination of the excised biopsies.

Supplement: Supplementary file 1 — Supplementary Material 1. The histopathological examination of the excised biopsies. [file 13256_2024_4657_MOESM1_ESM.docx]
